# Progress in Ophthalmology

**Published:** 1902-04-26

**Authors:** 


### PROGRESS IN OPHTHALMOLOGY.

**Relation of Gonorrhœa to Disease of the Eye** (excluding pur. ophthal.).—At the annual meeting of the British Medical Association, 1901, Mr. J. B. Lawford[1] read a paper on the above subject. To prevent misconception he definitely gave notice that gonorrhœal ophthalmia was to be entirely omitted from the discussion. At the beginning of his paper he quoted Professer Osler as follows:—Gonorrhœa is one of the most widespread of infectious diseases. As a cause of ill-health and disability the gonococcus occupies a position of the very first rank among its fellows. While the local lesion is too often thought to be trifling, in its singular obstinacy, in the possibility of permanent sexual damage to the individual, and still more in the "grisly troop" which may follow in its train, gonorrhœal infection does not fall far short of syphilis in importance. Having taken this statement for his text, he proceeds. Although the gonococcus had been demonstrated in the blood, in fluids within the joints, in exudation around joints, in the endocardium and other tissues, he was not aware that it had ever been detected in the eyeball, except in cases of gonorrhœal ophthalmia in which perforation had occurred. The ocular affections which are known to occur in association with gonorrhœa (exclusive of the conjunctivitis due to direct inoculation by the gonococcus) are a form of conjunctival inflammation, scleritis and episcleritis, iritis, and iridocyclitis, neuroretinitis. Suppurative keratitis has been described in cases of a severe pyæmic character. Iritis is probably the best known of these, although conjunctivitis is said to occur with greater frequency. Like all the remote manifestations of gonorrhœa, the above-mentioned lesions are much more common in the male sex. There are few statistics to guide one as to the frequency of ocular lesions, but Fournier met with ocular troubles 15 times in 39 cases. Gonorrhœal iritis in a large number of cases occurs concomitantly with arthritis. There seemed no doubt that its onset may precede that of joint inflammation, or that iritis may be present in cases where there are no joint complications. Fournier stated that in a series of cases observed by him, the joints and the eyes were not usually affected simultaneously. These two manifestations of gonorrhœa occur not only concomitantly in many instances, but also possess certain characteristics in common. They are both prone to relapse, and they leave behind them a certain vulnerability of the tissues which may, and often does, persist for a very long period. An eye which has been attacked by gonorrhœal inflammation and a knee-joint which has been the seat of gonorrhœal arthritis are liable to recurrences of inflammation without any fresh gonorrhœal infection. This liability may last for years. It had been suggested that gonorrhœa might be the cause of iritis developing for the first time long after all local and general signs of gonorrhœa had disappeared—the writer was very sceptical on this point. Scleritis and episcleritis of gonorrhœal origin, or at least associated with gonorrhœa, are much less common than iritis, but like the latter were usually met with in patients with joint inflammation and share the clinical features of iritis so far as intractability and liability to relapse are concerned. Among the less common eye affections attributed to the poison of gonorrhœa were retinitis, apparently of thrombotic character, and neuro-retinitis. Galezowski had suggested that the cases of retinitis were instances of thrombosis of retinal arteries due to an obliterating endarteritis, set up by an accumulation of gonococci in the vessels. Dr. Darier, of Paris, in the discussion which followed the paper, thought that these ocular and other complications followed the rough treatment of the urethritis with rupture of the mucous membrane or epithelium, and that they were due to a general infection. To this Mr. Cargill rejoined that if this were true, they should be between Scylla and Charybdis in scarifying the excessively chemotic conjunctiva of a gonorrhœl ophthalmia.

[1] Brit. Med. Jour., Nov. 2, 1901.

